# Project greener anaesthesia

**DOI:** 10.1038/s43856-023-00260-6

**Published:** 2023-03-18

## Abstract

Today is Global Recycling Day. All medical practices and disciplines face sustainability issues and there is a huge focus in the UK on minimising the environmental effects of medicine. Here we speak to Dr Daniel Lake about ‘Project Greener Anaesthesia’, which aims to do just that for Anaesthetics.

**Figure:**
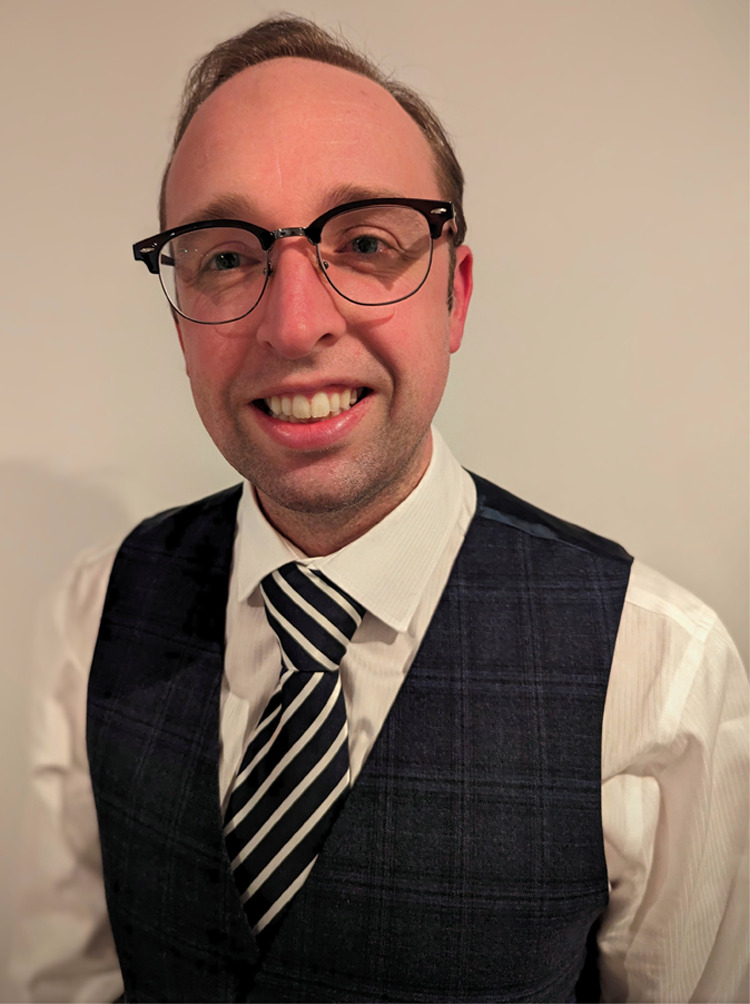
Daniel Lake

Dr Daniel Lake is an Anaesthetic and Intensive Care Consultant in the UK, working at Dartford and Gravesham National Health Services (NHS) Trust – an organisational unit within the NHS of England and Wales. Here, he has an additional role as Clinical Liaison to the Estates Team. The Estates Team are a senior management team for the Hospital that are responsible for all buildings and infrastructure. Daniel studied medicine at University College London, then did rotations through East Midlands, London and Kent hospitals. As well as a passion for Anaesthetics and Intensive Care, he feels that having a good understanding of how the hospital infrastructure works is key to maximising the quality of patient care. Though not an engineer, prior to medical school and during his studies he also worked for a heating and ventilation engineering consultancy company, which gave him experience in surveying, mapping, and troubleshooting issues with commercial ventilation systems. This experience, plus a love of physics, led him to being one of the team members to ensure that his hospital’s oxygen supply was able to cope with demands during the height of the COVID-19 Pandemic. This relationship with the Estates team led to the NHS Trust developing a new role of Clinical Liaison to try and ensure that key clinical aspects are not missed during planning and development of new hospital projects. NHS England, the government body primarily responsible for all NHS Trusts, released the Greener NHS initiative which has multiple aims to improve sustainability and reduce pollution from UK-based healthcare. It has a fundamental goal of the NHS delivering net zero carbon emissions by 2040. To implement this initiative each Trust has been advised to develop an NHS Green Plan, which has resulted in Daniel’s role - developing and driving forward sustainability projects across the trust, making hospital and patient services more environmentally-friendly.

Why is the anaesthetics medical specialty so detrimental for the environment?

Each hospital speciality has its own particular sustainability issues, but these have only come to light due to the positive remodelling of how we think about the services we deliver and their impact on the environment. Environmental factors have been highlighted in all walks of life by the mainstream press giving more time to people like Greta Thunberg and events such as the United Nations Climate Change Conference. This shift has resulted in environmental impact starting to play a part in decision-making in corporate or healthcare settings. It has even sparked debates in anaesthetic conferences about the environmental impact being discussed during the patient consenting process for medical treatments, for example the pollution aspect of analgesic choices for labouring women. Anaesthetics as a speciality has been targeted in the NHS England Green Plan as one of the key specialities where changes can be made. This is not only due to the huge volume of waste generated per anaesthetic but also, currently, the most frequently used anaesthetic technique requires the use of halogenated inhalational anaesthetic agents, which are a significant greenhouse gas. Out of the halogenated inhalational anaesthetic agents, Sevoflurane and Desflurane are the two most commonly used in the UK. Both these agents are potentially toxic to staff and so the National Institute for Occupational Safety and Health recommended exposure levels of less than 2ppm. Sevoflurane was traditionally thought to be more expensive than Desflurane but this argument is less valid now that both are off-patent. Desflurane has approximately one-third the potency of Sevoflurane, which means a higher partial pressure has to be delivered to achieve anaesthesia, yet it has the clinical benefits of much faster onset and offset (wakeup) times. Desflurane was originally taught as having the negative aspects of being pungent smelling and an airway irritant, making it very difficult to use for gaseous induction of anaesthesia. However, in the last year its environmental impact has been more widely discussed. Desflurane has 2500-times the warming effect of carbon dioxide and therefore a 1-hour anaesthetic with Desflurane is the equivalent to 30-60 Kg of carbon dioxide (the equivalent of driving 200-400 km). Given this huge carbon equivalent polluting effect, the use of Desflurane has been addressed directly by NHS England in the Greener NHS agenda. The other reason this is a key area of medicine to target for environmental gains is that halogenated inhalational anaesthetic agents are not the only way to perform an anaesthetic procedure and therefore there are alternatives already available such as Total Intravenous Anaesthesia (TIVA) and regional anaesthesia.

You have set up a programme called ‘Project Greener Anaesthesia’. How did that come about?

As mentioned, NHS England issued a Greener NHS agenda, which requests individual NHS Trusts to develop a Green Plan to meet the target of NHS net zero carbon emissions by 2040. Our trust created a Sustainability Steering Group led by the Estates team, which I sat on to offer clinical input. Given the high profile of Anaesthesia in the NHS Green Agenda and the significant contribution to greenhouse gases from Anaesthesia, I felt it wise to create a sub-committee with the vested parties involved. The committee of Project Greener Anaesthesia (Project GA) is made up of doctors, nurses, operating department practitioners (allied health professionals), hospital managers and the estates team. This mixture of clinical and non-clinical roles is key, in my opinion, to being able to rapidly implement change. Commonly in the past, clinicians have tried, and failed - or met obstruction, to implement service changes and this is simply due to not involving the correct non-clinical staff, which can be easily done in very large organisations. Not only did we formulate projects from the committee, but we also surveyed all staff working in the operating theatres for sustainability ideas. The response to this was very encouraging, as it not only highlighted project ideas that the committee was unaware of, but it also showed the huge amount of interest and support there was to make the service we provide as environmentally-friendly as possible.

What is next for Project GA?

We currently have 8 individual projects in place, ranging from the implementation of recycling greater than 95% of our anaesthetic room waste, to implementing digital systems, to providing paperless patient care pathways. There are multiple other projects, which have been submitted to the committee and are being reviewed for their viability. The other aspect of reducing the greenhouse emissions of anaesthesia is also reducing staff exposure to harmful agents. Nitrous oxide, an anaesthetic and analgesic agent used in theatres and labour ward is a large contributor to our carbon footprint. This is also a harmful agent if staff experience prolonged exposure as it can lead to vitamin B12 depletion, potentially resulting in cognitive and neuronal damage, memory loss, and other lasting effects. We at Project GA have developed a Nitrous oxide task force to work closely with representatives of the midwifery staff to provide a solution to the environmental issue and mitigate their risk. We are tackling the project with short-term solutions such as staff Nitrous Oxide exposure monitoring and location rotation, and more long-term, definitive solutions, such as nitrous oxide scrubbing. Scrubbing is the removal of the polluting nitric compounds from the collected air stream and converting to inert substances. There are multiple ways of doing this but we believe Nitrous Oxide destruction units located centrally attached to the Air Handling Unit for the Labour Ward will be the most effective. The air handling unit deals with the air flow to and from the labour ward and therefore by directly scrubbing nitrous oxide from the extracted air, the pollutants are removed directly prior to venting the extracted air to the atmosphere.

What do you think is achievable on a national scale to reduce the effects of anaesthetics on climate change?

The bigger aspect of Project GA is the inter-trust/healthcare-provider team working. As we are all working towards the NHS England Greener Plan it is sensible that many of these problems and solutions are shared and that we should work together to solve these issues. With that in mind, a Kent and Medway Green working group for a wider area has been formed and there are others in existence across the United Kingdom to facilitate group thinking and working, so I hope Project GA can collaborate with other likeminded groups. Anaesthetic sustainability has been gaining traction in recent public forums such as Anaesthetic conferences and statements by the Royal College of Anaesthetists, yet without grassroot action these plans and ideas cannot be implemented. I envisage that by sharing ideas and solutions we can rapidly reduce NHS carbon footprint attributed to anaesthesia and help Trusts reach the Greener NHS aim of Net zero within the next twenty years.

